# More than a technical fix: The long journey from paper logbooks to e-portfolios

**DOI:** 10.3205/zma001878

**Published:** 2026-09-15

**Authors:** Harm Peters, Ylva Holzhausen

**Affiliations:** 1 Charité – Universitätsmedizin Berlin, Dean’s Office for Study Affairs, Dieter Scheffner Center for Medical Education and Educational Research, Berlin, Germany

## Editorial

Since 2013, German medical faculties have been required to structure training in the practical year through subject-specific logbooks. The intention was to make the objectives of final-year education transparent, establish common expectations across clinical training sites, and provide students and supervisors with a framework for the activities and procedures that should form part of the placement. In this respect, logbooks have helped to make the intended curriculum more visible in a highly distributed phase of medical education.

Yet an intended curriculum does not automatically become educational practice. The study by Gerstacker and colleagues illustrates this gap in otorhinolaryngology. A substantial proportion of students and supervisors reported that they did not use a logbook at all. Nevertheless, most respondents favoured a digital version, and many students who rarely used the paper-based format expected to use it more frequently if it were available electronically [1]. While digitalisation may improve accessibility and potentially increase use, the authors stress that educational value depends on more than a change of medium and call for an interactive digital logbook with clear learning objectives. This raises a broader question: How can a logbook evolve into a developmental e-portfolio that becomes part of clinical learning rather than merely an electronic record of completed activities? The distinction lies in the educational purpose. Traditional logbooks primarily record whether predefined activities were completed. In contrast, an e-portfolio should connect evidence over time and use it to guide further learning and development. It should open a conversation rather than simply closing an item. In this editorial, we address this question by considering four interconnected perspectives on implementing an e-portfolio: a shared EPA framework, students’ individual learning pathways, supervisors’ educational practices, and faculties’ use of portfolio data for curricular learning.

At Charité, we recently introduced an EPA-based e-portfolio for the practical year, across the university campuses and its affiliated teaching hospitals [2]. Our experience since then suggests that the move from a paper-based logbook to an e-portfolio will likely be a long journey. For such a change to become implemented consistently across wards and training sites, students and supervisors need a shared understanding and language for professional activities, supervision, and development.

In the German context, the EPA framework in the Graduation Profile of the National Competence-Based Catalogue of Learning Objectives for Medicine (NKLM) can provide such a shared language and conceptual framework for the practical year [3]. EPAs describe authentic units of professional practice and connect performance with the level of supervision required. Their underlying logic is developmental. Students do not suddenly “have” or “not have” an EPA. Through observation, active participation, feedback, and practice, students can perform defined professional activities with progressively greater independence and decreasing levels of supervision.

A shared language and conceptual framework, however, are only the first step. If the developmental logic of the NKLM is to shape learning across the many sites of the practical year, it needs to be translated into a shared infrastructure through which expectations, observations, and student´s progress can be communicated. Within the Medical Faculty Association, German medical faculties are currently discussing the development of a national e-portfolio for the practical year. Such a portfolio could establish a shared understanding: which professional activities students are expected to perform at which level of supervision by the end of the practical year. It would provide clinical supervisors with the same frame of reference, helping them to create appropriate learning opportunities for their students, observe performance, and transfer responsibility safely. In today’s clinical environment – characterised by rotating clinical teams, multiple hospitals, and many supervisors – this shared understanding should not be taken for granted. 

At the same time, a common framework should not imply identical learning pathways. Our experience with an EPA-based e-portfolio in the practical year at Charité shows why [2]. At the beginning of the placement, students reported markedly different levels of prior practical experience. Some had repeatedly performed certain activities with considerable independence; while others had had little opportunity even to attempt them. Structured documentation within an EPA framework makes these differences visible at a point when they can still inform learning. Supervisors can adapt task allocation and supervision, while students can identify priorities for an individualised learning plan. In this way, the e-portfolio turns retrospective documentation into prospective learning support. It not only records what has happened; it informs what should happen next. It can prompt students to seek specific learning opportunities, help supervisors decide when direct observation is necessary, and enable both to review whether independence is increasing over time. Its value lies in connecting data with educational action.

The value of these data extends beyond the individual student to the medical faculty level [2]. Paper-based logbooks usually remain collections of individual entries. By aggregating data across students, placements, and training sites, an e-portfolio can reveal how the practical year curriculum actually works in practice. It can provide insight into which professional activities students perform, at what levels of supervision, where experiences vary, and where the intended curriculum is not consistently realised. This enables the medical faculties to respond through targeted curricular adjustments. The e-portfolio thereby connects two learning processes: students learn from experience, and faculties learn from students’ documented experiences how the curriculum they provide is enacted in practice.

An e-portfolio will not implement itself. To realise its educational potential, it needs to be designed around clinical workflows, not the other way around. Students need orientation and ongoing support in using feedback and portfolio data to shape and steer their own EPA-based learning trajectories in busy clinical workplaces. Supervising physicians need training and support in direct observation, providing feedback, and making entrustment decisions. Implementation should therefore be accompanied by concise, just-in-time faculty development, integrated into everyday workflows: brief EPA descriptions before observation, clarification of supervision levels when rating performance, prompts for narrative feedback, and suggestions for agreeing on the next learning step. The e-portfolio should also enable observations and assessments of student’s performance to be recorded close to the clinical encounter and fed directly into each student's longitudinal record. The technical implementation may take months; cultural integration into daily clinical practice will likely take years.

The move from paper-based logbooks to a nationally shared e-portfolio for the practical year in Germany is likely to be a long journey: from documentation to development, from implicit expectations to a shared curricular understanding, and from individual records to institutional learning. Such an e-portfolio would constitute a curricular infrastructure that needs to become part of everyday clinical practice. Its promise will be realised only when it helps medical students grow into professional responsibility, supports clinical supervisors in integrating students into patient care, and enables medical faculties to understand how their curricula actually work in practice. Achieving this is more than a technical fix.

## Authors’ ORCIDs

- Harm Peters: [0000-0003-1441-7512]
- Ylva Holzhausen: [0000-0001-8710-8257]

## Competing interests

The authors declare that they have no competing interests. 
